# Impact of Computed Tomography-to-Angiography Interval Time on Outcomes of Transarterial Embolization in Post-Traumatic Bleeding: A Retrospective Observational Study

**DOI:** 10.3390/jpm15110528

**Published:** 2025-11-02

**Authors:** Claudio Carrubba, Francesco Giurazza, Fabio Corvino, Federico Capozzoli, Raffaella Niola

**Affiliations:** 1 Vascular and Interventional Radiology Department, Cardarelli Hospital, Via Antonio Cardarelli 9, 80131 Naples, Italy; claudio.carrubba@aocardarelli.it (C.C.); fabio.corvino@aocardarelli.it (F.C.); raffaella.niola@aocardarelli.it (R.N.); 2 Department of Advanced Biomedical Sciences, University of Naples “Federico II”, 80131 Naples, Italy; federico.capozzoli@unina.it

**Keywords:** multiple trauma, embolization, therapeutic, tomography, X-ray computed, interventional radiology, hemostasis, lactates

## Abstract

**Background/Objectives:** Transarterial embolization nowadays has a pivotal role in non-operative management strategies of post-traumatic bleeding. Timely control of hemorrhage is critical in trauma care; however, the impact of procedural timing remains underexplored. This single-center study, conducted at a Level II trauma center with 24/7 interventional radiology coverage, evaluated the influence of interval time on embolization outcomes in post-traumatic bleeding patients. **Methods:** In this retrospective study, 182 trauma patients who underwent embolization between June 2020 and June 2025 were analyzed. Patients were stratified by CT-to-angiography interval time (≤1 h [early, n = 46] and >1 h [delayed, n = 136]). Hemodynamic parameters, laboratory values, transfusion needs, and outcomes were compared and adjusted for baseline differences. **Results:** Early group patients showed more severe baseline physiology, including hypotension, higher lactates, and lower hemoglobin. No significant differences were found in mortality (2.9% vs. 2.5%), hospital stay (18.7 ± 26.1 vs. 18.1 ± 22.2 days), or transfusion requirements. Embolizations within one hour from CT were associated with significant lactate reduction at 24 h in univariate analysis (*p* = 0.039), but this was not confirmed in multivariate analysis. Re-embolization (8.7% vs. 1.5%, *p* = 0.036) and surgical rescue (13.0% vs. 3.7%, *p* = 0.033) rates were more frequent in the early group. **Conclusions:** Early embolization improves metabolic parameters in post-traumatic bleeding, especially in patients with greater baseline severity of injuries. These findings support prioritization of early embolization and structured interventional radiology networks for timely procedures. A personalized approach according to baseline injury is required.

## 1. Introduction

Hemorrhage accounts for 30–40% of trauma-related deaths, with 33–56% of these deaths occurring in the prehospital phase—a clear indicator that rapid hemorrhage control is critical to improving survival outcomes [1,2,3,4]. Prompt and effective intervention is crucial to improve survival and minimize long-term complications in patients with post-traumatic bleeding [5]. Among hemostatic strategies for trauma patients, transarterial embolization (TAE) has become a cornerstone of non-operative management (NOM), particularly in cases of visceral parenchymal injuries or pelvic fractures [6,7,8]; in these scenarios, a personalized approach aiming to maximize the clinical benefits and minimizing the operative invasiveness is essential.

In U.S. national trauma databases, conventional angiography is performed in approximately 0.53% of trauma admissions, with 55.4% of these patients undergoing TAE. Large multicenter cohorts—both European and U.S.—report technical success rates close to 100% and clinical success rates of ~91–93% for embolization in traumatic hemorrhage, with rebleeding episodes effectively managed through re-embolization. Although the overall incidence of TAE in trauma is still low, it represents a pivotal component of non-operative management in patients with major injury (ISS ≥ 16), consistently achieving high hemostatic efficacy and contributing to reduced need for surgical rescue [9,10,11,12].

Early angiographic intervention is increasingly recognized as a key component in optimizing outcomes for polytraumatized patients with active bleeding confirmed by computed tomography (CT) [13]. Recommendations from Interventional Radiology (IR) scientific societies emphasize the need for fast management protocols in trauma care, including 24 h availability of multidetector computed tomography (MDCT), robust multidisciplinary collaboration, and immediate access to angiography with 24/7 IR staff coverage, ideally able to act by 1 h from diagnosis [14,15]. Similarly, the 2023 World Society of Emergency Surgery (WSES) guidelines highlight the importance of early TAE in patients with active arterial bleeding and imaging evidence of ongoing hemorrhage [16].

Multiple series have linked shorter CT-to-angiography (or door-to-embolization) times with improved outcomes in traumatic hemorrhage [17,18]. In complex pelvic fractures, shorter interval time was associated with better 24 h survival and clinical outcomes [19]. A nationwide analysis showed that delays to TAE were associated with higher adjusted 30-day mortality (up to 17% when embolization occurred at 150–180 min, vs. 0% within 0–30 min) [20]. U.S. cohorts similarly report that every hour of delay to TAE increased mortality risk, and that achieving median time <90 min correlated with lower in-hospital mortality in patients with pelvic fracture and shock [21]. Beyond patient-level findings, a prospective survey of U.S. Level I trauma centers documented substantial variability in IR availability and setup times, underscoring system-level contributors to delay [22].

However, most studies are retrospective, prone to confounding by indication (sicker patients triaged to faster TAE), and use non-uniform time metrics (emergency department access-to-TAE, CT-to-TAE, door-to-embolization) and heterogeneous definitions of clinical success, which limit cross-study comparability and causal inference [23].

This study has been conducted in a Level II trauma center with 24/7 IR coverage and aims to evaluate the impact of CT-to-angiography interval time on technical and clinical outcomes of TAE in post-traumatic bleeding patients.

## 2. Materials and Methods

### 2.1. Study Design

This monocentric retrospective observational study was conducted at a regional Level II trauma center equipped with a 24/7 IR team with active on-site coverage. The institution houses a dedicated Trauma Center Unit within the Department of Emergency and Critical Care. The Radiology Unit, which serves as the hub for imaging emergencies, performs over 20,000 emergency CT scans annually, ensuring rapid access to advanced diagnostic imaging for trauma patients.

Electronic medical records were reviewed over the last five years (June 2020–June 2025).

Inclusion criteria were as follows: traumatic event; CT scan showing active arterial bleeding; angiographic embolization; availability of clinical and laboratory data for up to 30 days of follow-up.

Exclusion criteria were: (I) surgical interventions performed before embolization; (II) angiography performed more than 12 h after the index CT; (III) no evidence of active bleeding at CT.

Patients were stratified into two groups according to the time elapsed between CT acquisition and angiography: early group (≤1 h) and delayed group (>1 h). The CT-to-angiography interval was calculated in minutes, from the scout timestamp recorded in the Picture Archiving and Communication System (PACS) to the arterial puncture time documented in the operative log.

### 2.2. Data Collection

All relevant data were extracted from the institutional electronic medical records and the Picture Archiving and Communication System (PACS) by two independent investigators, using a standardized data collection form. Discrepancies were resolved by consensus with a third reviewer. Demographic, clinical, laboratory, imaging, and procedural variables were cross-checked across multiple sources (ED records, operative logs, radiology reports) to ensure accuracy. Missing or ambiguous information was verified by reviewing original imaging or source documents. To minimize information bias, all variables were defined a priori according to established trauma research standards, and outcome assessment was performed blinded to group allocation.

The following data were collected:

• Injury Severity Score (ISS), calculated according to the Abbreviated Injury Scale (AIS) 2015 update, for all included patients;
• Admission and post-procedural hemodynamic parameters. At Emergency Department (ED) admission, hemodynamic parameters were recorded, including systolic blood pressure (SBP), diastolic blood pressure (DBP), mean arterial pressure (MAP), heart rate (HR), and Shock Index (SI; calculated as HR(beats per minute)/SBP(mmHg));
• Baseline and 24 h hemoglobin and lactate levels;
• Post-embolization transfusion requirements, massive transfusion protocol (MTP) activation, and overall transfusion balance. Massive transfusion protocol (MTP) was defined as the transfusion of ≥10 units of packed red blood cells within the first 24 h after admission;
• Timing intervals, including ED-to-CT and CT-to-angiography intervals;
• CT and angiographic findings, classifying bleeding sites as pelvic, abdominal, thoracic, or other.
• Re-interventions: re-embolization was defined as any repeated TAE for persistent or recurrent hemorrhage during the index hospitalization. Surgical rescue included damage-control laparotomy (e.g., splenectomy, bowel repair/resection), pelvic external fixation with/without preperitoneal packing (PPP), and other hemostatic procedures performed after the index TAE. Injury categories were predefined as pelvic, abdominal (solid organ or hollow viscus), thoracic, or others. For surgical rescue, multiple procedures could be performed in the same patient; therefore, totals may exceed the number of patients.

Cases with missing primary outcome data (lactates or hemoglobin at 24 h) were excluded from the respective analyses; no imputation was performed.

### 2.3. Patient Selection

**Figure 1:**
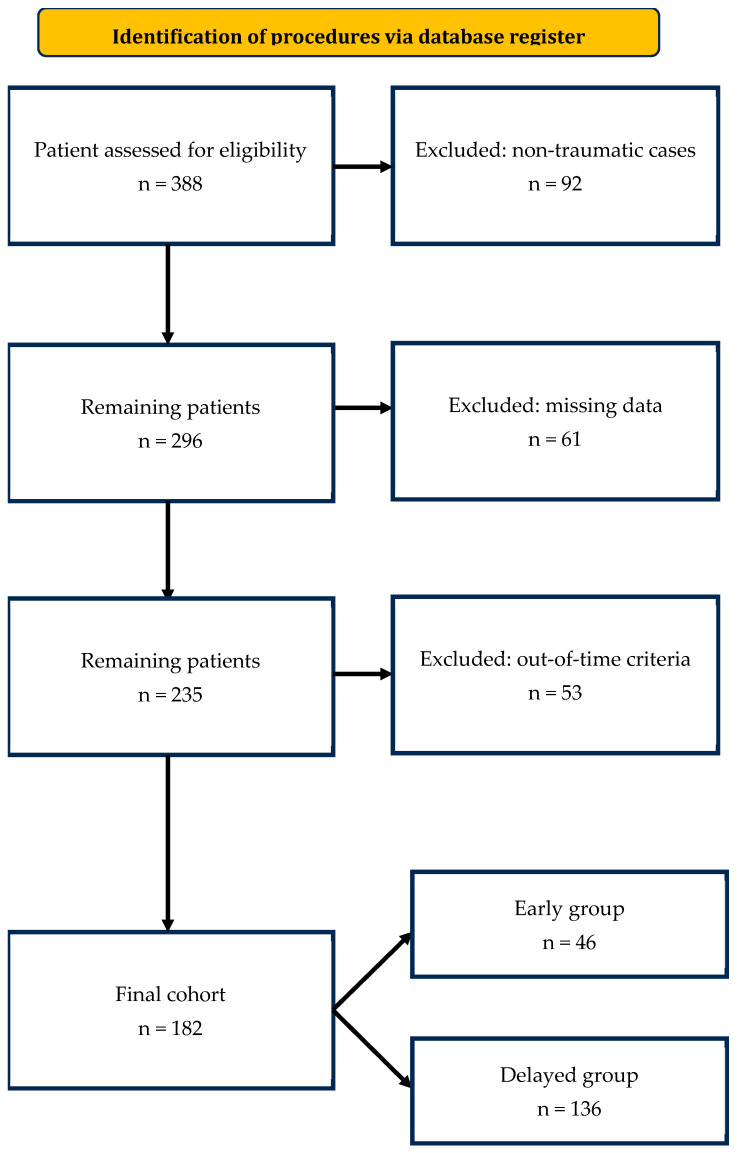
**Patient selection flowchart according to STROBE guidelines.** Flow diagram illustrating patient selection, reasons for exclusion, and final allocation to the early (≤1 h) and delayed (>1 h) intervention groups.

The initial cohort included 388 consecutive patients presenting with hemorrhagic conditions and undergoing angiographic procedures. A total of 92 patients were excluded due to non-traumatic etiologies, 61 were excluded because of incomplete clinical or laboratory data, and 53 did not meet the predefined time criteria for CT-to-angiography interval. The final study cohort consisted of 182 patients, stratified into two groups according to the time from initial diagnostic CT to the start of transarterial embolization: early group (≤1 h, *n* = 46) and delayed group (>1 h, *n* = 136). The patient selection process and reasons for exclusion are summarized in Figure 1, following STROBE recommendations.

### 2.4. Outcome Measures

Primary outcome was defined as the absolute change in serum lactates (mmol/L) and hemoglobin (g/dL) from baseline to 24 h after angiographic embolization. Secondary endpoints included in-hospital mortality, length of hospital stay (LOS), transfusion requirements, reintervention rates (re-embolization or surgical rescue), and procedure-related complications, evaluated according to the timing of the TAE (< or >1 h).

### 2.5. Procedural Details

Upon ED arrival, patients underwent initial clinical evaluation and were prioritized for contrast-enhanced CT in case of suspected bleeding. Based on imaging findings, the ED physician formally requested the IR on site to review scans and assess the indication for embolization. A multidisciplinary consensus—including surgeons, anesthesiologists, emergency physicians, and interventional radiologists—determined the need for embolization. Patients were then moved to the angiography suite, staffed by at least one interventional radiologist, an anesthesiologist, two nurses, and a radiographer.

**Figure 2:**
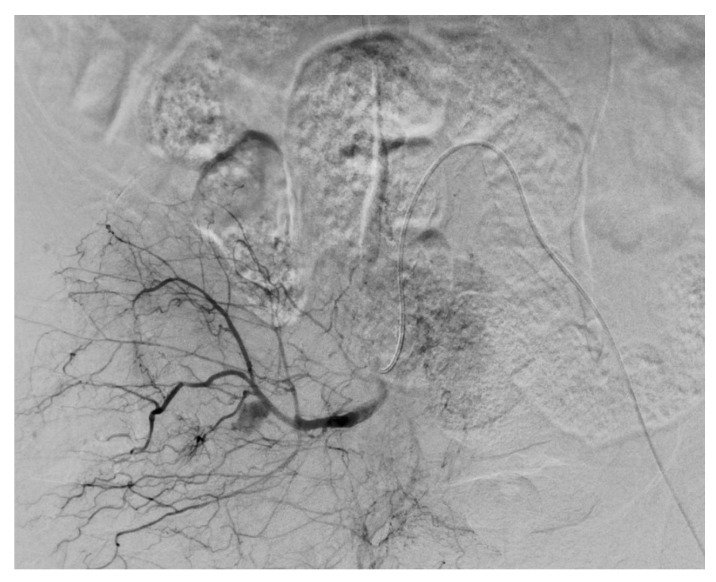

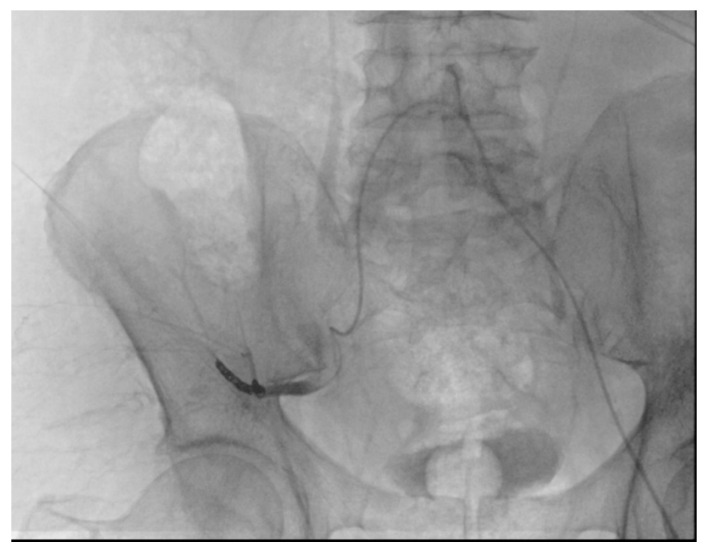
**Active arterial blush from the epigastric artery treated with coils.** Selective angiographic image showing active extravasation successfully embolized using microcoils.

All procedures were performed urgently via a common femoral approach by interventional radiologists with at least five years of experience in post-traumatic bleeding interventions, using modern angiography systems (Artis Zee^®^, Siemens, Erlangen, Germany). Superselective embolization with a microcatheter was attempted whenever possible to minimize non-target embolizations. The choice of embolic agent was based on anatomical location, angiographic findings, coagulation status and operator preference: n-butyl cyanoacrylate glue was preferred in cases of coagulopathy, whereas in other cases the choice depended on operator preference (Figure 2).

Vascular closure devices were used to reduce procedural time. Post-procedure, patients were admitted to the emergency surgery or intensive care units according to clinical status. Technical success was defined as complete embolization of the target vessel. Clinical success was defined as improvement of hemodynamic status, cessation of transfusions and amelioration of laboratory parameters.

All complications were classified according to the CIRSE classification system [24].

### 2.6. Statistical Analysis

Statistical analysis was performed using SPSS software (version 28.0, IBM, Armonk, NY, USA). The Shapiro–Wilk test was used to assess the normality of continuous variables. Normally distributed data are presented as mean ± standard deviation (SD), whereas non-normally distributed data are reported as median and interquartile range (IQR). Categorical variables are expressed as counts and percentages. Effect estimates for continuous and categorical outcomes were calculated with corresponding 95% confidence intervals. For continuous variables, confidence intervals were derived using the t-distribution; for proportions, the exact binomial method was applied. All multivariable model results are reported as regression coefficients or odds ratios (OR) with 95% confidence intervals.

Inter-group comparisons were performed using the independent-samples *t*-test for normally distributed variables and the Mann–Whitney U test otherwise. Chi-square or Fisher’s exact tests were applied for categorical data. Multivariable linear regression was used for continuous outcomes (Δlactates, Δhemoglobin, LOS), while logistic regression was used for mortality, adjusting for age, baseline values, intubation status, transfusion requirements, and Injury Severity Score (ISS). Covariates for multivariable models were pre-specified based on clinical relevance (age, baseline lactates and hemoglobin, intubation status, ISS) and/or a univariable association with *p* < 0.10, with model parsimony enforced given event counts. Significant baseline differences between groups (e.g., admission hemoglobin, lactates, ISS) were included as covariates in the multivariable models to adjust for potential confounding.

Before inclusion in multivariable models, all covariates were assessed for multicollinearity using the variance inflation factor (VIF), with a threshold of <5 considered acceptable. No significant collinearity was detected. Effect estimates for continuous and categorical outcomes were calculated with corresponding 95% confidence intervals. Confidence intervals for continuous variables were derived using the t-distribution, while proportions were calculated using the exact binomial method. A *p*-value < 0.05 was considered statistically significant.

All consecutive eligible patients over the 5-year study period were included, yielding a sample of 182 cases. A post hoc power calculation indicated that, for continuous outcomes (Δlactates, Δhemoglobin), the study had 80% power to detect a standardized mean difference (Cohen’s d) of approximately 0.48 between groups at α = 0.05. For the observed difference in re-embolization rates (8.7% vs. 1.5%), the post hoc power was approximately 61%.

## 3. Results

### 3.1. Patient Characteristics

In this study 182 trauma patients were considered: 46 patients (25.3%) received TAE within 1 h from CT acquisition (early group), while 136 (74.7%) underwent embolization after more than 1 h (delayed group).

**Figure 3:**
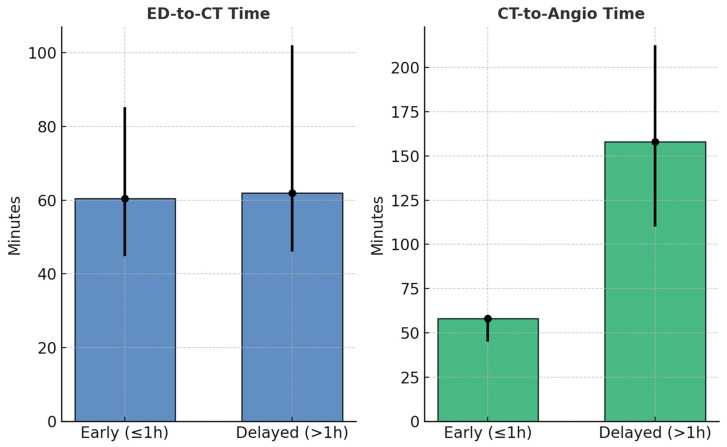
**Workflow timing comparison between early and delayed groups.** Comparison of (**right**) CT-to-angiography and (**left**) ED-to-CT time intervals between early (≤1 h) and delayed (>1 h) angiography groups. Data are shown as median values with interquartile ranges (IQR).

CT-to-angiography times were significantly shorter in the early group (58 min [IQR, 45–60] vs. 158 min [IQR, 110–212.5]; *p* < 0.001; 95% CI for difference: −111 to −86 min). ED-to-CT intervals slightly favored the early group (60.5 min [IQR, 44.8–85.3] vs. 62.0 min [IQR, 46.0–102.0]; *p* = 0.26; 95% CI −9.5 to 6.5 min), suggesting a more streamlined workflow (Figure 3).

The mean age was comparable between groups (51 years [IQR, 30.5–67] early vs. 58 years [IQR, 36.5–75.5] delayed), with a predominance of males (73.9% vs. 66.9%). The pelvis was the most frequently treated anatomical region (58.8%), followed by the abdomen (18.7%) and thorax (8.2%). Less common sites included isolated injuries to the back, limbs, neck, or combined regions (14.3%), reflecting the heterogeneous distribution of traumatic hemorrhages.

Baseline differences in admission concerning hemoglobin, lactates, and ISS favored the delayed group; these variables were adjusted in multivariable analyses. At ED admission, mean arterial pressure was slightly lower in the early group (80 ± 13 mmHg [95% CI 76.1–83.9] vs. 83 ± 14 mmHg [95% CI 80.6–85.4]; *p* = 0.18), while heart rate and SI were marginally higher (98 ± 18 bpm [95% CI 92.7–103.3] vs. 95 ± 17 bpm [95% CI 92.1–97.9]; *p* = 0.32, and 0.92 ± 0.20 [95% CI 0.86–0.98] vs. 0.85 ± 0.17 [95% CI 0.82–0.88]; *p* = 0.10, respectively). Systolic and diastolic blood pressure were comparable between groups (110 ± 18 mmHg vs. 113 ± 20 mmHg, *p* = 0.34; 68 ± 12 mmHg vs. 70 ± 11 mmHg, *p* = 0.32). The mean Injury Severity Score (ISS) was significantly higher in the early group (24 ± 8; 95% CI 21.7–26.3) compared to the delayed group (18 ± 6; 95% CI 17.0–19.0; *p* < 0.0001), indicating more severe trauma at baseline (Table 1). Despite higher baseline ISS, no patient in either group required MTP activation; the median transfusion requirement was 1 unit of packed red blood cells (PRBC), with interquartile range 0–2.

### 3.2. Laboratory and Hemodynamic Outcomes

At presentation, patients in the early group demonstrated worse clinical conditions, including hypotension, tachycardia, higher rates of pre-procedural intubation, and elevated baseline lactate levels (2.2 mmol/L [IQR, 1.48–3.13] vs. 1.9 mmol/L [IQR, 1.3–2.65]; *p* = 0.46; 95% CI −0.18 to 0.78), with the highest recorded values being 3.13 and 2.6 mmol/L, respectively.

**Figure 4:**
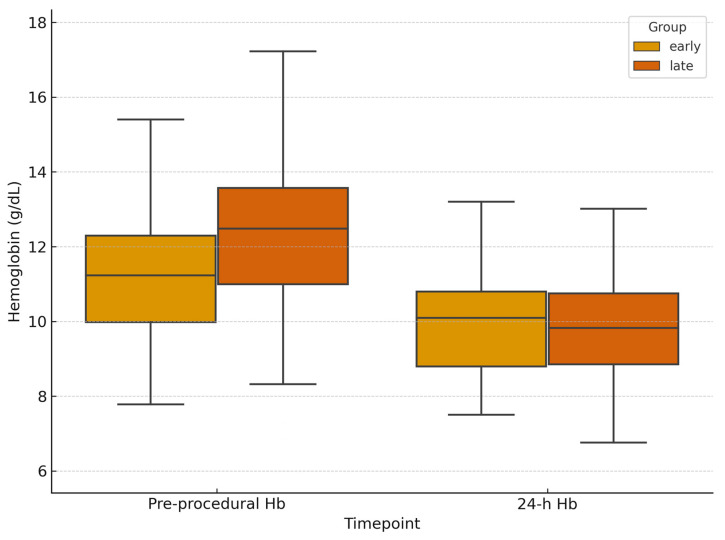
**Hemoglobin levels before and 24 h after embolization.** Box plots showing hemoglobin variation from baseline to 24 h post-procedure, stratified by treatment timing.

**Figure 5:**
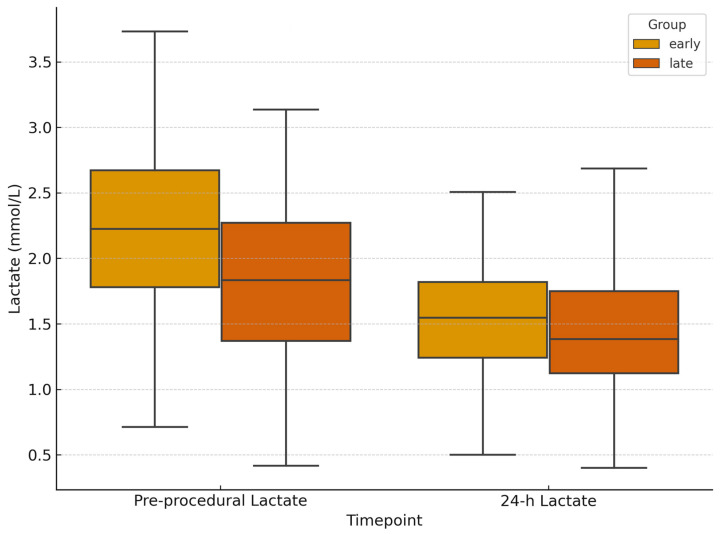
**Lactate levels before and 24 h after embolization.** Box plots depicting the reduction in serum lactate levels over 24 h post-embolization in early versus delayed intervention groups.

**Figure 6:**
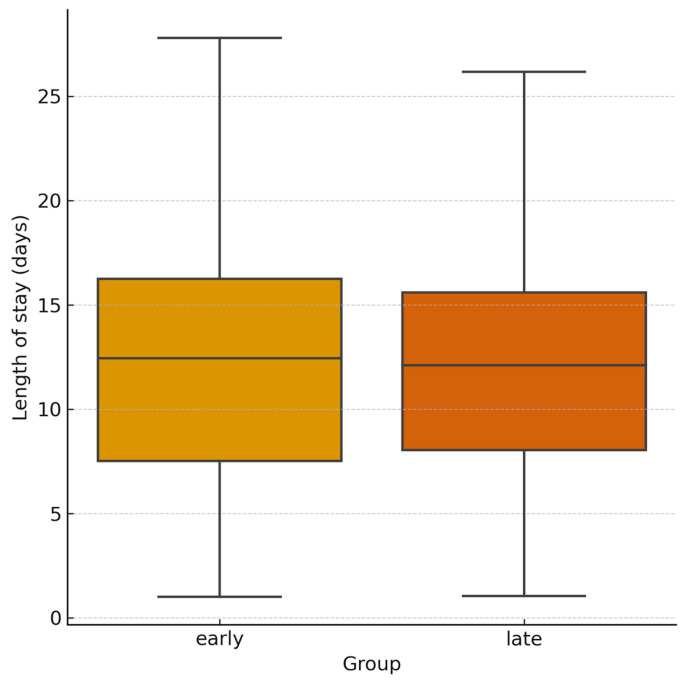
**Length of hospital stay by treatment group.** Box plot comparing total hospital stay duration between early and delayed embolization cohorts.

Pre-procedural hemoglobin was lower in the early group (11.7 g/dL [IQR, 9.68–13.17] vs. 12.3 g/dL [IQR, 10.23–13.58]; *p* = 0.13; 95% CI −1.38 to 0.18), consistent with more severe injuries. At 24 h follow-up, hemoglobin levels were similar between the two groups (9.85 g/dL [IQR, 8.68–10.78] vs. 9.8 g/dL [IQR, 8.78–11.2]; Figure 4). Hemoglobin variation was lower in the early group (−0.95 g/dL; 95% CI −1.42 to −0.48) vs. −1.60 g/dL (95% CI −1.94 to −1.26), although the difference was not statistically significant (*p* = 0.12; 95% CI for difference −0.12 to 1.42). Lactate levels decreased in both cohorts. Median 24 h lactate levels were 1.5 mmol/L [IQR, 1.05–2.48] in the early group and 1.4 mmol/L [IQR, 1.0–1.9] in the delayed group, with a non-significant greater reduction in the early group (Δlactates (24 h-baseline): −0.7 mmol/L; 95% CI −0.95 to −0.45) vs. −0.5 mmol/L (95% CI −0.62 to −0.38); *p* = 0.79; 95% CI for difference −0.19 to 0.59 (Figure 5). Post-procedural transfusion requirements were comparable (1 unit [IQR, 0–2] in both groups), totaling 69 and 158 PRBC units in the early and delayed groups, respectively. No massive transfusion protocol (MTP) activations occurred. Technical success was 100% in both groups. Clinical success was higher in the delayed group with early group requiring higher re-embolization rates (8.7% vs. 1.5%; *p* = 0.036; 95% CI for OR 1.10–12.45) and more frequent surgical rescue treatment (13.0% vs. 3.7%; *p* = 0.033; 95% CI for OR 1.13–9.85). Despite baseline differences, long term clinical outcomes were similar across the groups. In-hospital mortality was 2.9% in the early group and 2.5% in the delayed group (*p* = 0.40; 95% CI for OR 0.25–9.12). Mean length of stay was also comparable (12 days [IQR, 6–25] vs. 11 days [IQR, 6–20]; *p* = 0.89; 95% CI for difference −2.5 to 3.5; Figure 6; Table 2).

### 3.3. Re-Interventions

Re-embolization occurred in 4/46 patients (8.7%) in the early group and in 2/136 (1.5%) in the delayed group (*p* = 0.036). Surgical rescue was required in 6/46 (13.0%) early vs. 5/136 (3.7%) delayed patients (*p* = 0.033). The distribution of surgical procedures is reported in Table 3. In the early group, the most frequent procedures were preperitoneal pelvic packing (PPP) and pelvic fixation (each performed in three patients, 6.5%), followed by damage-control laparotomy with temporary abdominal closure (two patients, 4.3%). Splenectomy, bowel repair/resection, and hepatorrhaphy/hepatic packing were less common (each performed in one patient, 2.2%). In the delayed group, splenectomy and PPP were each performed in two patients (1.5%), whereas pelvic fixation, damage-control laparotomy, bowel repair/resection, and nephrectomy/renorrhaphy were each performed in one patient (0.7%). Several patients underwent more than one procedure, resulting in a higher total number of procedures than patients treated.

### 3.4. Procedure-Related Complications

Minor complications (CIRSE Grade 1–2) occurred in five early-treated patients (10.9%) and eleven delayed-treated patients (8.1%; *p* = 0.56; 95% CI for OR 0.32–2.98): post-embolization syndrome (four and five cases, respectively) and access-site hematomas (one and six cases, respectively). Major complications rate (CIRSE Grade 3–4) were 2.2% in the early group (one pseudoaneurysm at the puncture site); 2.9% in the delayed group (two abscesses/tissue necroses requiring drainage or prolonged antibiotic therapy and two access-site pseudoaneurysms requiring endovascular management) (*p* = 1.00; 95% CI for OR 0.05–6.52).

### 3.5. Multivariable Analysis

**Figure 7:**
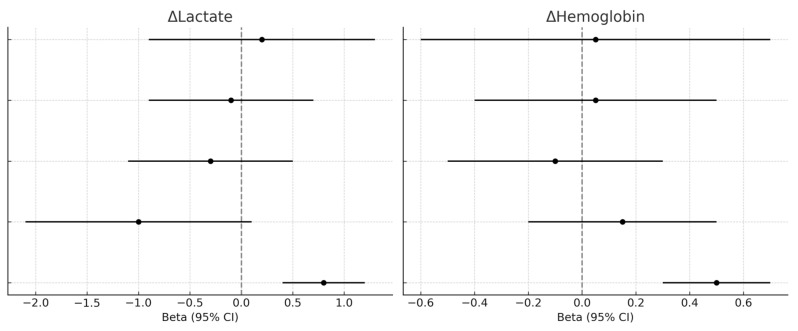
**Multivariable regression models for hemoglobin and lactate changes.** Forest plots displaying adjusted beta coefficients and confidence intervals for predictors of 24 h hemoglobin and lactate variation.

Multivariable models adjusted for baseline hemoglobin, lactate levels, and ISS did not identify early angiographic intervention (≤1 h) as an independent predictor of 24 h changes in hemoglobin (β = 0.05; 95% CI −0.55 to 0.65; *p* = 0.86), lactates (β = 0.23; 95% CI −0.87 to 1.33; *p* = 0.68), or in-hospital mortality (OR = 2.80; 95% CI 0.77–10.15; *p* = 0.12). Baseline lactates emerged as a significant predictor of mortality (OR = 1.38; 95% CI 1.10–1.72; *p* = 0.006). Sensitivity analyses confirmed these findings, suggesting that although early intervention may facilitate prompt hemostatic stabilization, its measurable impact on laboratory and clinical outcomes is largely modulated by baseline severity and resuscitation needs (Figure 7).

## 4. Discussion

This study evaluated the impact of procedural timing on outcomes of TAE in post-traumatic bleeding patients, comparing interventions performed < or >1 h from initial diagnostic CT. Patients treated early presented with more severe clinical profiles, characterized by hemodynamic instability, lower hemoglobin levels, higher lactates at baseline, and a significantly higher ISS (mean 24 ± 8 vs. 18 ± 6; *p* < 0.001). Admission hemodynamics showed non-significant differences between groups, with a trend toward lower MAP and higher SI in the early cohort, consistent with their overall greater baseline severity. TAE ≤60 min was associated with early metabolic improvement (lactates), without independent effect after adjustment and no significant differences in mortality, hospital stay, or transfusions. Despite this, early TAE was associated with a significant lactate reduction and less hemoglobin decrease at 24 h, while in-hospital mortality, transfusion requirements, and length of stay remained comparable to those treated later. Technical success was similar in both groups. In context of personalized medicine, these findings are consistent with previous studies emphasizing the value of early embolization. In our cohort, patients with hemodynamic anomalies and metabolic compromise were prioritized for rapid intervention, aligning with observations by DuanMu et al., who demonstrated reduced mortality, shorter hospitalization, and decreased transfusion needs when pelvic embolization was performed early [18]. Similarly, O’Connell et al. reported significant mortality benefits for pelvic TAE performed within 90 min [21], while other studies identified a linear association between longer door-to-angiography times and higher in-hospital mortality [22,23,24]. Yamamoto et al. [25] recently reported in their nationwide retrospective analysis that in trauma patients, immediate angiography (<30 min) was associated with lower in-hospital mortality and fewer non operative management failures. Although our data do not show a direct mortality advantage for early TAE, similar survival values among the two groups suggest that early intervention may mitigate the deleterious effects of delayed hemorrhage control described in prior reports. Although MTP activation was not required in either group, several patients presented with clinical signs of severe trauma, including hypotension, elevated shock index, metabolic acidosis, and a need for pre-procedural intubation. The estimated ISS was markedly higher in the early group (24 ± 8 vs. 18 ± 6), confirming greater baseline severity despite similar transfusion volumes. We believe that rapid bleeding control in a high-volume trauma center with 24/7 IR availability may have contributed to limiting massive transfusion needs and improving survival even in hemodynamically unstable patients. Nevertheless, the relatively low transfusion volumes observed should be acknowledged as a limitation when extrapolating these results to settings with higher baseline injury severity or delayed access to definitive hemostasis.

From a metabolic perspective, lactate values are widely recognized as a surrogate marker of tissue hypoperfusion and a predictor of adverse trauma outcomes [26,27]. In our cohort, admission lactate levels were only moderately elevated, corresponding to mild hyperlactatemia in most laboratory reference ranges. This finding should be interpreted in light of our institutional workflow: measurements were obtained upon ED arrival, often after pre-hospital resuscitation and during early in-hospital stabilization, which may have partially normalized circulating levels before the index measurement. Moreover, early access to definitive hemorrhage control—frequently within the first hour from imaging—may have further limited lactate accumulation. Despite being in the range of mild hyperlactatemia, values above 2 mmol/L have been associated with increased morbidity and mortality in trauma patients. Bou Chebl et al., in a large European cohort of critically ill emergency department patients, demonstrated that admission lactate values between 2 and 4 mmol/L were independently associated with significantly higher in-hospital mortality compared to values < 2 mmol/L (12% vs. 2.7%, *p* < 0.01) [28]. Therefore, even mild lactate elevations retain prognostic significance in a personalized trauma care, supporting the relevance of our findings. The significant lactate reduction observed in early-treated patients aligns with findings by Sadrzadeh et al., who emphasized early lactate changes as a prognostic indicator and a trigger for urgent hemorrhage control [29]. Higher re-embolization and surgical rescue rates in the early group likely reflect the greater complexity of injuries prioritized for urgent intervention rather than procedural inadequacy. The distribution of rescue procedures supports this interpretation: PPP and pelvic fixation predominated in the early cohort, accompanied by damage-control laparotomy and selected solid-organ resections (e.g., splenectomy, hepatic packing), indicating high-grade pelvic and abdominal injuries requiring staged hemorrhage control. Several patients underwent multiple procedures, underscoring the multifocal nature of bleeding and the need for a multimodal damage-control strategy (Table 3). In contrast to studies on solid-organ injuries where TAE has been associated with ischemic complications [30,31], complication rates in our cohort remained overall low. This discrepancy may be related to anatomical targets, embolization techniques, and the predominance of pelvic hemorrhage, highlighting the safety of TAE even in urgent settings. These findings are in line with European guidelines [32], recommending prompt hemorrhage control to prevent coagulopathy and systemic complications, emphasizing early activation of IR teams. The comparable clinical outcomes and faster metabolic stabilization in our early group highlight the importance of streamlined protocols enabling rapid transition from imaging to definitive hemostasis. Certainly, having an IR team constantly on site eases the application of this quick workflow. These observations underscore the systemic need for organized fast-track interventional radiology networks. The latest CIRSE Clinical Practice Survey (2024) reported that only ~50% of European radiology departments maintain a structured IR unit, with fewer than 20% having dedicated IR inpatient beds and less than 35% running day-case wards [33]. Variability in infrastructure and workforce shortages hinder the widespread implementation of early TAE. Continuous quality improvement processes together with personalized approaches are essential to maintain institutional readiness for urgent interventions [34]. Future prospective multicenter studies are warranted to better delineate the independent effect of early TAE on outcomes beyond metabolic parameters, particularly in settings with higher baseline injury severity and less immediate access to interventional radiology.

This study presents some limitations. It was conducted in a single high-volume trauma center with continuous IR availability, which limits the reproduction of the findings to other healthcare settings. Specific biases related to treatment timing should be acknowledged, including potential confounding by indication and possible measurement inaccuracies in determining the CT-to-IR interval. External validity may also be limited, as our findings may not be directly applicable to institutions without 24/7 interventional radiology coverage or to those operating under different organizational models, such as direct-to-angio pathways or hybrid emergency/angiography rooms. In our adjusted analyses, covariate selection was based on clinically relevant baseline variables available in our dataset; however, the absence of standardized trauma severity scores such as Revised Trauma Score (RTS) or Trauma and Injury Severity Score (TRISS), in addition to ISS, should be considered a relevant limitation. The retrospective design, although reflective of real-world practice, precludes causal inference, and residual confounding from unmeasured variables cannot be excluded despite multivariable adjustments; however, prospective randomization would be ethically unacceptable in this context. Moreover, the lack of a demonstrated independent effect of early embolization on laboratory or survival outcomes likely reflects the predominant influence of baseline injury severity on prognosis. Nevertheless, early angiographic intervention remains a cornerstone of trauma damage control, and its benefits may extend beyond immediate laboratory endpoints, reinforcing its role in the comprehensive management of complex trauma.

## 5. Conclusions

In this retrospective cohort, TAE performed within 60 min from diagnostic CT was associated with earlier metabolic improvement, as reflected by greater lactates reduction. However, this association did not remain independently significant after multivariate adjustment, and no statistically significant differences were observed in mortality, hospital stay, or transfusion requirements compared to those patients treated with TAE after more than 60 min from CT acquisition. While early intervention may contribute to faster correction of hypoperfusion, its impact on hard outcomes was not demonstrated in this study. A personalized approach according to baseline injury is required. These findings highlight the need for multicenter prospective studies with adjustments for standardized severity variables to confirm and expand these results.

## Figures and Tables

**Table 1:** **Baseline injury severity and admission hemodynamic parameters in early (≤1 h) and delayed (>1 h) embolization groups.** Values are expressed as mean ± standard deviation (SD) with 95% confidence intervals (CI). *p*-values refer to between-group comparisons (independent-samples *t*-test). ISS: Injury Severity Score; MAP: mean arterial pressure; SBP: systolic blood pressure; DBP: diastolic blood pressure; HR: heart rate; SI: Shock Index (calculated as HR/SBP).

Parameter	Early (n = 46)	95% CI	Delayed (n = 136)	95% CI	*p*-Value
Injury Severity Score (ISS)	24 ± 8	21.7–26.3	18 ± 6	17.0–19.0	<0.0001
Mean arterial pressure (mmHg)	80 ± 13	76.1–83.9	83 ± 14	80.6–85.4	0.18
Systolic BP (mmHg)	110 ± 18	—	113 ± 20	—	0.34
Diastolic BP (mmHg)	68 ± 12	—	70 ± 11	—	0.32
Heart rate (bpm)	98 ± 18	92.7–103.3	95 ± 17	92.1–97.9	0.32
Shock Index (HR/SBP)	0.92 ± 0.20	0.86–0.98	0.85 ± 0.17	0.82–0.88	0.10

**Table 2:** **Comparison of clinical, laboratory, and procedural outcomes between early and delayed angiography groups.** Statistical comparison of baseline characteristics, laboratory values, transfusion needs, clinical outcomes, and complication rates stratified by timing of angiographic embolization.

Variable	Early Group (≤1 h)	Delayed Group (>1 h)	*p*-Value	95% CI
CT-to-angiography time (min, median [IQR])	58 [45–60]	158 [110–212.5]	<0.001	−111 to −86 min
ED-to-CT time (min, median [IQR])	60.5 [44.8–85.3]	62.0 [46.0–102.0]	0.26	−9.5 to 6.5 min
Age (years, median [IQR])	51 [30.5–67]	58 [36.5–75.5]	-	-
Male sex (%)	73.9%	66.9%	-	-
Baseline hemoglobin (g/dL, median [IQR])	11.7 [9.68–13.17]	12.3 [10.23–13.58]	0.13	−1.38 to 0.18
Baseline lactates (mmol/L, median [IQR])	2.2 [1.48–3.13]	1.9 [1.3–2.65]	0.46	−0.18 to 0.78
ΔHemoglobin at 24 h (g/dL)	−0.95 (95% CI −1.42 to −0.48)	−1.60 (95% CI −1.94 to −1.26)	0.12	−0.12 to 1.42
ΔLactates at 24 h (mmol/L)	−0.7 (95% CI −0.95 to −0.45)	−0.5 (95% CI −0.62 to −0.38)	0.79	−0.19 to 0.59
PRBC transfusion post-procedure (units, median [IQR])	1 [0–2]	1 [0–2]	-	-
Total PRBC transfused (units)	69	158	n.a.	-
Massive transfusion protocol activation	0	0	-	-
Technical success (%)	100%	100%	-	-
Re-embolization (%)	8.7%	1.5%	0.036	OR 1.10–12.45
Surgical rescue (%)	13.0%	3.7%	0.033	OR 1.13–9.85
In-hospital mortality (%)	2.9%	2.5%	0.40	OR 0.25–9.12
Length of hospital stay (days, median [IQR])	12 [6–25]	11 [6–20]	0.89	−2.5 to 3.5
Minor complications (CIRSE Grade 1–2, %)	10.9%	8.1%	0.56	OR 0.32–2.98
Major complications (CIRSE Grade 3–4, %)	2.2%	2.9%	1.00	OR 0.05–6.52

Data are expressed as median [interquartile range] for continuous variables and as number (percentage) for categorical variables, unless otherwise specified. Reported statistics include *p*-values and 95% confidence intervals (CI) for between-group differences or odds ratios (OR), as appropriate.

**Table 3:** Post-TAE re-interventions: re-embolization and surgical rescue procedures by group.

Procedure	Early (n = 46)	Delayed (n = 136)
Preperitoneal pelvic packing (PPP)	3 (6.5%)	2 (1.5%)
Pelvic fixation/SI screws/C-clamp	3 (6.5%)	1 (0.7%)
Damage-control laparotomy + temporary abdominal closure	2 (4.3%)	1 (0.7%)
Splenectomy	1 (2.2%)	2 (1.5%)
Bowel repair/resection	1 (2.2%)	1 (0.7%)
Hepatorrhaphy/hepatic packing	1 (2.2%)	0 (0.0%)
Nephrectomy/renorrhaphy	0 (0.0%)	1 (0.7%)

Surgical rescue procedures performed after index TAE. Values are n (% of patients in each group). Legend: Percentages are calculated using the total number of patients in each group as the denominator. Patients could undergo more than one procedure.

## Data Availability

The data presented in this study are available on request from the corresponding author.

## Abbreviations

The following abbreviations are used in this manuscript:

TAE	Transarterial Embolization
CT	Computed Tomography
NOM	Non-Operative Management
ISS	Injury Severity Score
IR	Interventional Radiology
MDCT	Multidetector Computed Tomography
WSES	World Society of Emergency Surgery
ED	Emergency Department
PACS	Picture Archiving and Communication System
SBP	Systolic Blood Pressure
DBP	Diastolic Blood Pressure
MAP	Mean Arterial Pressure
HR	Heart Rate
SI	Shock Index
MTP	Massive Transfusion Protocol
PRBC	Packed Red Blood Cells
PPP	Preperitoneal Packing
LOS	Length of Stay
RTS	Revised Trauma Score
TRISS	Trauma and Injury Severity Score
